# “We have this, with my husband, we live in harmony”: exploring the gendered decision-making matrix for malaria prevention and treatment in Nampula Province, Mozambique

**DOI:** 10.1186/s12936-020-03198-5

**Published:** 2020-03-30

**Authors:** Zoe Jane-Lara Hildon, Maria Escorcio-Ymayo, Rose Zulliger, Rosario Arias de Aramburú, Nan Lewicky, Hunter Harig, Jose Braz Chidassicua, Carol Underwood, Liliana Pinto, Maria Elena Figueroa

**Affiliations:** 1 Johns Hopkins Bloomberg School of Public Health, Center for Communication Programs, 111 Market Place, Suite 310, Baltimore, MD 21202 USA; 2 Saw Swee Hock School of Public Health, National University of Singapore, Tahir Foundation Building, 12 Science Drive 2, Level 09-03J, Singapore, 117549 Singapore; 3 U.S. President’s Malaria Initiative, Centers for Disease Control and Prevention, Maputo, Mozambique; 4 Johns Hopkins Bloomberg School of Public Health, Center for Communication Programs, Maputo, Mozambique

**Keywords:** Community dialogues, Gender roles and decision making, Communication, Malaria prevention

## Abstract

**Background:**

Conceptualizing gender dynamics and ways of bridging entrenched gender roles will contribute to better health promotion, policy and planning. Such processes are explored in relation to malaria in Mozambique.

**Methods:**

A multi-method, qualitative study using focus group discussions (FGDs) and in-depth interviews (IDIs) explored the perspectives of community members, leaders and stakeholders on malaria. The study was conducted in Nampula Province, in an intervention district for the Tchova Tchova Stop Malaria (TTSM) gender-sensitive community dialogues, and in a non-intervention district.

**Results:**

Participants (n = 106) took part in six FGDs and five IDIs in each district. Those exposed to TTSM commonly stated that the programme influenced more equalitarian gender roles, attitudes and uptake of protective malaria-related practices. These positive changes occurred within the context of an observed, gendered decision-making matrix, which aligns inward- or outward-facing decisions with malaria prevention or treatment. Decisions more dependent on male or elder sanctioning at community level are outward-facing decisions, while decisions falling within women’s domain at household level are inward-facing decisions. Related to prevention, using bed nets was largely an inward-facing prevention decision for women, who were generally tasked with hanging, washing and making nets usable. Net purchase and appropriation for malaria prevention (rather than for instance for fishing) was men’s prerogative. Regular net use was associated with sleeping together more regularly, bringing couples closer. Attending antenatal care to access intermittent preventive treatment during pregnancy was often an outward-facing prevention decision, under the purview of older, influential women and ultimately needing sanctioning by men. With respect to seeking care for malaria symptoms, women typically sought help from traditional healers first. This inward-facing treatment decision was within their control, in contrast to the frequently transport-dependent, outward-facing decision to attend a health facility. Sharing decisions was described as a feature of a “harmonious household,” something that was said to be encouraged by the TTSM intervention and that was both lived and aspirational.

**Conclusions:**

TTSM community dialogues helped communication on both interpersonal (couple) and community levels, ultimately encouraging malaria-related behaviours. Leveraging ways of bringing men and women together to share decision making will improve malaria intervention success.

## Background

Although important progress in malaria prevention and treatment has been achieved, African regions still account for 92% of the global burden of malaria cases and 93% of malaria deaths worldwide [1]. Pregnant women and children under the age of 5 years remain the most vulnerable to malaria, especially when inadequate nutritional conditions further weaken their capacity to fight the disease [1]. Vulnerability to malaria is therefore driven by complex epidemiological, environmental and social factors that are tied to specific settings.

Mozambique is among the low-income countries that have achieved Millennium Development Goal 4 target set in 2000 to reduce under-5 mortality by over two-thirds [2]. Yet, malaria remains an endemic disease in Mozambique [3], where there were an estimated 10,025,823 cases and approximately 14,713 deaths in 2017 [1].

Malaria is a leading cause of maternal and child mortality in Mozambique. Between 2007 and 2011, verbal autopsy data revealed that malaria was responsible for 23% of all maternal deaths [4]. Among children aged 1–4, malaria led to 51% of all deaths [5], while parasitaemia also disproportionally affected children living in rural areas (47%) compared to those in urban areas (19%) [6].

There are three widely implemented protective behavioural interventions for malaria prevention: (1) consistent use of an insecticide-treated net (ITN); (2) antenatal care (ANC) visits administering intermittent preventive treatment in pregnancy (IPTp); and, (3) use of indoor residual spraying. When prevention fails, prompt effective confirmatory diagnosis and treatment at a health facility or from a community health worker is advised. Context and cultural experiences may facilitate or constrain uptake of these behaviours. Consequently, understanding decision-making processes and autonomy is central to understanding related patterns of these behaviours as well as ways of influencing them.

For instance, Nhatave [7] found that gender-related factors, including the influence of a partner, family members and social norms, affect a woman’s decision to seek prompt health care. In addition, multiple factors including education level, perceived disease severity, distance to facilities, costs of health care, socio-economic status, and experience when seeking health care are all known to influence care seeking [8, 9].

Mozambique is classified as having high access to public facilities (> 65%) [10] and in rural Zambézia, Mozambique, 72% of caregivers reported seeking advice for fever in children under 5, with 91% reportedly doing so at a health facility [11]. Nationally, while 66% of households owned at least one ITN, only 48% of children under 5 and 52% of pregnant women reported sleeping under an ITN in households that had at least one of these [6].

In addition, the proportion of women in Mozambique with at least one ANC visit is high at 93%, yet the percentage with four or more visits is only 55% [6]. Although IPTp has been implemented as routine practice and is expected to be provided during women’s ANC visits [12], it is concerning that the proportion of women who received two or more doses of IPTp during their last pregnancy increased from only 20% in 2011 [13] to 34% in 2015 [6].

### *Tchova Tchova* Stop Malaria community dialogue programme

The current study sought to explore key protective malaria-related behaviours, unpacking in particular the decision-making process in relation to an existing intervention conducted in the Nampula Province of Mozambique. Beginning in 2016, the Health Communication Capacity Collaborative (HC3) undertook the *Tchova Tchova* Stop Malaria (TTSM) gender-sensitive community dialogue programme, which was implemented in Zambézia, Nampula and Tete provinces. TTSM was implemented in selected districts based on local malaria burden in consultation with the provincial department of health.

TTSM was adapted from a successful HIV programme in Mozambique, *Tchova Tchova Histórias de Vida: Diálogos Comunitários*, meaning Push Forward Life Stories: Community Dialogues, first launched in Mozambique in 2008 [14, 15], and the *Prevenção Activa e Comunicação Para Todos* or Active Prevention and Communication for All project in 2010 [16, 17]. Central to each of these programmes lies the participatory, community-level, bottom-up approach, which encourages normative changes and changes in attitudes through public dialoguing and community engagement on sensitive topics.

TTSM community dialogue sessions were implemented by trained facilitators from the community itself. The sessions were delivered with the aim of engaging men and women, and especially couples, as well as neighbours and wider community members in discussions on how to create more equal gender dynamics. This included improving communication and sharing of decisions, even sharing of domestic duties between men and women in the home, while also encouraging the use of products and services to combat malaria. Fun and interactive activities and video materials were used to this effect (http://www.infosaude.gov.mz/wp-content/uploads/2019/09/Pacote-comunitario-tchova–tchova-stop-malária.pdf). In sum, the programme emphasized community mobilization to improve prevention and treatment seeking for malaria with a concurrent aim of addressing gender inequalities.

### Exploring gender dynamics

Research across African settings such as Kenya [18], Uganda [19] and Tanzania [20] has shown that gender dynamics significantly influence the uptake of measures to prevent, treat and control malaria. In Mozambique, a country which scores near the bottom of the Human Development and Gender Inequality Index [21], gender inequities and implicit social hierarchies that favour men influence uptake of malaria-related behaviours. This is because women in Mozambique are known to shoulder the majority of the burden that comes with running a household and caring duties [22].

Most of the malaria interventions at community or facility levels in Mozambique have been designed to reach women, often through multiple ANC visits, and children under 5 and pregnant women are consistently most likely users of ITNs [23]. A recent qualitative study in Zambézia Province found that barriers to ANC included gender inequality in decision-making, and the responsibility for pregnancy being seen to have to be largely shouldered by the woman [24]. In addition, the study highlighted community beliefs that ANC uptake, particularly if supported by a male partner, reflected a woman’s HIV-positive status. In Sofala Province, it was found that women who reported intimate partner violence had lower odds of achieving at least one ANC visit, four or more ANCs visits and of receiving ANC from skilled personnel [25].

Since interventions have not typically sensitized men in the importance of ongoing ANC visits and targeted them with malaria messaging, even though men have long been known to play a critical role in decision making about health care [26], it is time to consider how this oversight may have limited the success of malaria programmes. It can also be argued that, overall, female-centred programmes have missed the opportunity to promote strengthening couples’ shared decision making and to fully tackle reducing routes of transmission by excluding men.

The current analysis explores how dominant, patriarchally sanctioned hierarchical experiences and decision making shape the uptake of malaria-related behaviours in two rural districts in Nampula Province, Mozambique. The study also explores how participation in TTSM influenced such decision making. The results will inform future communication campaigns, including community-based strategies to improve malaria prevention and treatment.

## Methods

### Study aims

The study aims were to explore gender dynamics and processes in relation to the uptake of the following malaria-related practices: (1) Use and misuse of nets; (2) Pregnant women’s visits to health facilities for ANC/IPTp; (3) Fast, effective care seeking for fever at a health facility *versus* from a traditional healer. These behaviours were explored in relation to gender and decision making (in both districts) and the effect of TTSM (in the intervention district).

### Study setting

**Fig. 1:**
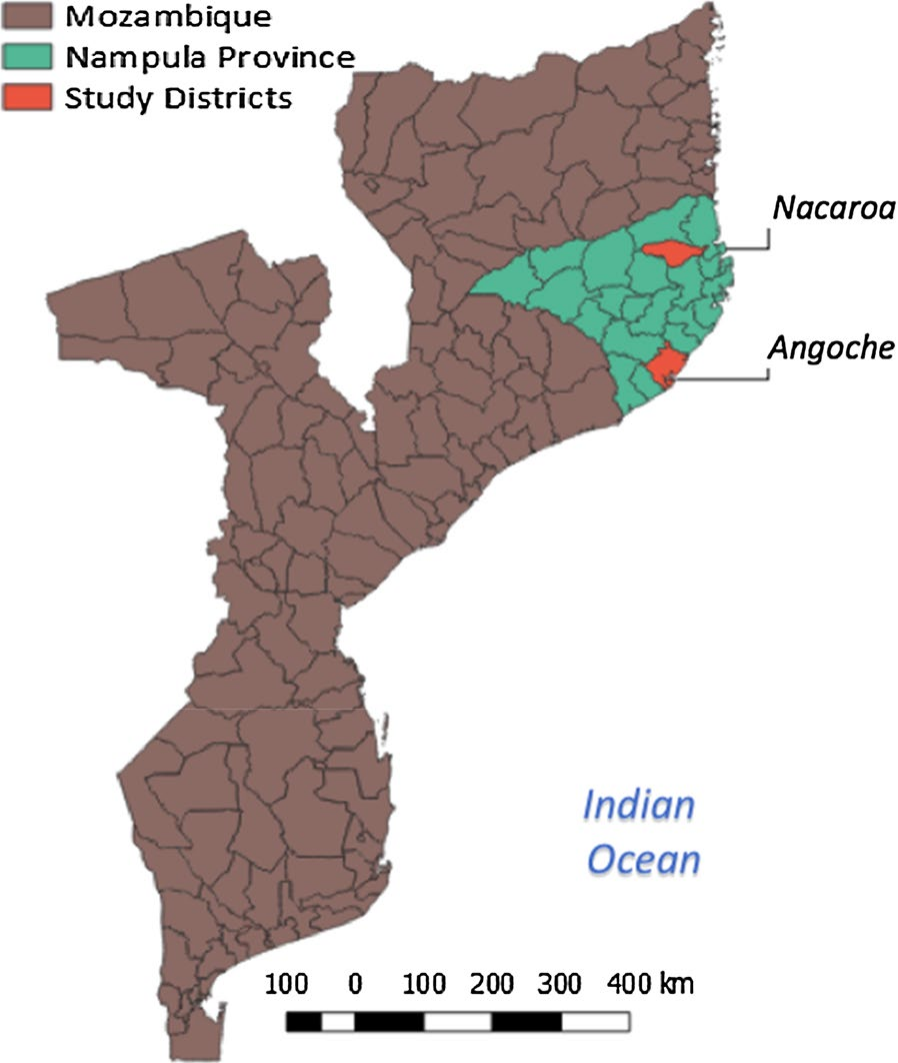
Map of Mozambique with Nampula Province highlighted, and study districts indicated

The study was conducted in two purposively selected rural districts in Nampula Province (Fig. 1): Angoche, the intervention district that participated in malaria community TTSM dialogues, and Nacaroa, the comparison, non-intervention district. Angoche is a coastal, peri-urban district (est. population = 399,092 in 2017) that is predominantly Muslim [27], with strong matrilineal influences. In contrast, Nacaroa is a rural district (est. population = 145,643 in 2017) close to the large port city of Nacala that is more Christian [28], with some matrilineal influences and households [29]. Nampula Province has one of the highest malaria prevalence rates in Mozambique (66%) [6] and is also the most populous province in the country, comprising 21% of the population [30]. The Mozambique Ministry of Health and the National Malaria Control Programme selected the province as the first province to be targeted for the implementation of the 2016/2017 national universal ITN distribution campaign to ensure all families have access to ITNs.

### Study design

Based on a social constructivist approach, this qualitative study used both focus group discussions (FGDs) and in-depth interviews (IDIs) to understand participants’ views and gendered malaria-related experiences. Using these two qualitative methods allowed for interactive as well as more in-depth exploration of gender-related decision making for malaria prevention and treatment.

### Study population

A purposive sample was selected from each district. The research team conducted six FGDs in each of the district, three among men and three among women. Each FGD included eight community member participants. Five IDIs with key influencers were also undertaken with community leaders, mothers-in-law, matrons (senior women leaders in communities), traditional healers, and traditional birth attendants (TBAs) in each district. The study population is described in Table 1.

**Table 1:** Study population sampled from Angoche and Nacaroa in Nampula Province

Study population	Method	Nampula Province		Totals
		Angoche (intervention)	Nacaroa (comparison)	
Men (20–49 years)	FGD	3	3	6 (48 people)
Women (18–44 years)	FGD	3	3	6 (48 people)
Community leaders (male)	IDI	1	1	2
Traditional healers (male)	IDI	1	1	2
Traditional birth attendants (female)	IDI	1	1	2
Mother-in-law (45–60 years)	IDI	1	1	2
Matrons (female)	IDI	1	1	2
Total FGDs		6 (48 people)	6 (48 people)	12 (96 people)
Total IDIs		5	5	10
	Total			106 people

The FGDs were conducted with equal numbers of men and women in the community (n = 48 each). However, community leaders and traditional healers included in the study were all men, and all matrons and TBAs were women. These gender assignations frame community actors’ roles and must be born in mind throughout data collection and analysis.

### Data collection instruments

This study used a semi-structured, paper-based topic guide for both the FGDs and IDIs to capture participants’ knowledge base, normative influences and gender roles in decision making. While knowledge and attitudes relating to each of the behavioural areas were considered, these are not the focus of the current study. Instead it focused on data elicited and coded around the following specific topics:

**Gender roles in decision-making with respect to malaria-related practices**

Information, decision-making, and performance of behaviours (contrasting all perspectives: men’s, women’s and community actors’)

- Information access, gender dynamics, and effects on decision making (in general)
- Use and misuse of nets
- Pregnant women going to the facility for ANC/IPTp
- Recognition of symptoms and decisions to get care

**Effects of the TTSM community dialogues** (perspectives from intervention site only)

- Information transmission and programme messaging that worked well/not so well in TTSM
- Changing views on men’s and women’s roles in TTSM
- Ways of being more concordant in decision making

As described above, perspectives on gender and decision-making were asked of all participants in relation to malaria-related behaviours of interest while topics relating explicitly to exploring the effects of the TTSM programme were asked of the programme participants only.

### Data collection procedures

Two data collection teams were created, one for each district, each with one team leader (MEY or JBC), one coordinator and three facilitators. Most of the data were collected in the local dialect, Makua, otherwise in Portuguese. The groups and interviews were run in the language chosen by the participants so that they were able to express themselves in the one that they were most comfortable with. Male and female facilitators were matched to participants where possible. All verbatim transcripts of the audio recordings were compiled in Portuguese by facilitators and approved by team leads. Written informed consent and permission to audio record was sought prior to IDIs and FGDs.

### Data cleaning and preparation

All data were audio recorded, transcribed and checked for quality and consistency. The team leads were both bilingual in English and Portuguese, and one lead additionally spoke Makua. This facilitated quality checks and checking for meaning against audio recordings, and also translation of written data, as elaborated below. To supplement the audio recordings of the IDIs and FGDs, field notes were taken by the facilitators and shared with the study coordinators and team leads. The field notes were systematically compiled by topic and stored with the verbatim transcripts.

The data preparation method was adapted from Halcomb and Davidson [31], and expanded notes were extracted from the field notes and verbatim transcripts and compiled in English by one bilingual analyst (MEY). The data were organized according to the specific topics listed above in ATLAS.ti for Mac (version 1.5.4). A non-Portuguese-speaking senior social scientist (ZJH) conducted quality checks of the expanded English notes on about one-quarter (n = 6) of the data, checking against the full verbatim transcripts, which were translated into English.

### Methods of analysis and reporting

This study used the framework analysis method [32, 33], indexing the data according to the specific topics listed above, while checking if new areas beyond those in the existing list emerged. No additional topics emerged. Two analysts indexed (MEY and ZJH) the data in ATLAS.ti, which were exported for charting and thematic coding into Microsoft Word files. Each topic was then manually coded, and emerging themes were summarized using salient, illustrative quotes. Thematic coding of the data was divided between the three team members (ZJH, MEY, RA), all trained in qualitative data analysis. At least 15% of the data were double-coded across sections and analysts reached a consensus on the meaning of the data. ZJH refined all the agreed analysis and finalized the reporting.

In the following sections, major themes are reported in **bold** and supporting sub-themes in *italics*. The more dominant themes are supported by a wider range of reported sub-themes. Where relevant that a consensus or a shared view was emerging by a majority this is indicated. Minority voices are also highlighted. The origin of quotes coming from the intervention site in Angoche (A) or the non-intervention site Nacaroa (N) are indicated accordingly. Analysis is reported according to the following structure: lived experiences of gender roles, the decision-making matrix, and effects of the TTSM community dialogues.

### Ethical review and approvals

The Johns Hopkins University institutional review board and the local research ethics committee in Mozambique (*Ministério da Saúde, Comité Nacional de Bioética para a Saúde*) reviewed and approved the study in March 2017. This study was also reviewed by the Centers for Disease Control and Prevention (CDC) and determined to be human subjects research with non-engagement by CDC staff.

## Results

### Lived experiences of gender roles

Data saturation was achieved in the synthesis and extraction of all major themes. Four major themes were identified in relation to the lived experiences of how gender roles influence malaria-related decision making. These relate to (1) **structure of the household** where the decision is made; expected (2) **gender power dynamics** within the couple when a decision is required; (3) **the degree to which men and women are receptive to information**; and, the concept of (4) a **harmonious household**, which was described as men and women being able to successfully dialogue, reach shared decisions, and take action together.

The **structure of the household** influenced malaria prevention and treatment. For instance, women-headed households had different decision-making structures than those headed by men, but sometimes still relied on male sanctioning. In such situations an uncle who lived nearby might be solicited as part of a decision-making process. He could also be deferred to as the key decision maker on important decisions in the woman-headed household.

In both study sites, irrespective of household structure, net use decisions, in particular, were often said to be *shared with women, mainly, “by default”.* That is, since women were the ones who hung nets, washed and repaired them, put the children to bed, and were left to organize the use of nets in the course of managing the household, they held decision-making authority in this realm:

*“Men say that they don’t have time (to help around the house), they need to be tending the farm”.* (N, women FGD, 02)

Women’s control rested at the household level, but overall, *husbands and older men usually said to be important in purchasing nets, using them properly in the first place (e.g., not as fishing nets), and accessing them and other types of care through health services.*

In certain instances, the decision-making power could occasionally *be shifted from the man to the mother*-*in*-*law, particularly if the extended family lived together or close by.* The mother-in-law was sometimes said to be responsible for helping decide when and with whom her pregnant daughter-in-law would go to ANC and whether her grandchildren with malaria symptoms would seek treatment at the health facility or from a traditional healer and follow traditional practices. Participants also mentioned that the mother-in-law would be the one who

*“takes her daughter*-*in*-*law, when pregnant, to ANC if her son is not around.”* (N, mother-in-law IDI).

In younger couples,

*“if the wife gets sick, the man goes and informs his mother (asking her permission) and if he has money, he goes to the health facility with his wife.”* (N, women FGD, 03)

In the matrilineal households in the intervention site, a participant also noted the following: *“Who motivates (the woman to seek ANC) when she is pregnant for the first time? It is her husband’s grandmother and her older sister*-*in*-*law.”* (A, mother-in-law IDI).

**Gender power dynamics** result from gender norms and expectations imposed by the community. *Men often referred to women as secondary to them men, describing them as simply “followers” of men, acting “like their shadow”.* Men explained that they therefore needed to take the leadership role to counter-balance women’s poor decision making. “*Bad” “female” decisions were attributed to women seeking treatment for malaria with a traditional healer* instead of going to a health facility despite the perception by male participants that it was a well-known fact that the facility setting was the option providing better care. Women, however, were quick to explain that these decisions are influenced by the lack of money for getting to the facility and the need to seek alternative transportation methods when it is far away. Women often mentioned that *decisions on spending any significant amount of money needed to be shared*.

In regard to **receptivity to information,***women were consistently described as more open than men*. Men were just as consistently described as less interested than women or disinterested altogether, as one traditional healer summarized it

*“mostly they (the men) scorn that information, only some follow it.”* (A, traditional healer IDI).

Pregnant women are also the ones described as attending weekly educational sessions with a traditional birth attendant or a nurse, not the men. *Men tended to describe themselves as more “intellectual” than women*, appearing more critical of intervention messages. One participant stated that “there is a war” to convince men to follow advice such as going with a woman to the health facility (A, matron IDI). For translating knowledge to action, men were said by some to need extra reinforcement to change their way of thinking, one example was given thus:

*“(before, when) we would get the nets, after getting home my husband would sell some; others he took to fishing. We had no nets, and (then) he got sick. We went to the hospital, did a test, and (found out he) had malaria. Then he understood.”* (A, women FGD, 03).

In addition, feedback both from community leaders and matrons also suggested that changing men’s minds in particular “is a battle”. These study participants noted that explicitly and repeatedly showing men the benefits of ascribed behaviours is needed to change them. Also, the perception that men were somehow more knowledgeable than women suggested that men would receive messages better “man to man”. That said, the study team identified a positive dynamic in a number of participants referring to aspiring to have a **harmonious household** which manifested as healthy interactive decision making between couples and greater gender equity, whereby men notably played a more supportive role helping in the home. In these instances, men were described as wanting to contribute, for example

*“They are involved in the process of reducing (their child’s) fever (through the use of a wet rag) and then take the child to (seek care).”* (N, men FGD, 02).

The notion of a harmonious household was referred to mostly by the female respondents in FGDs and IDIs, at both sites, and recognized as a cultural phenomenon. *Harmonious households* were viewed as a product of harmonious relationships, in which men have good feelings towards their partner and their children. A few men who had participated in TTSM noted that

“*if there is no collaboration, it is difficult to prevent malaria.” (A, men FGD, 01) and two are better than one. When there are two, then they can help each other.”* (A, men FGD, 01).

### The gendered decision-making matrix

**Fig. 2:**
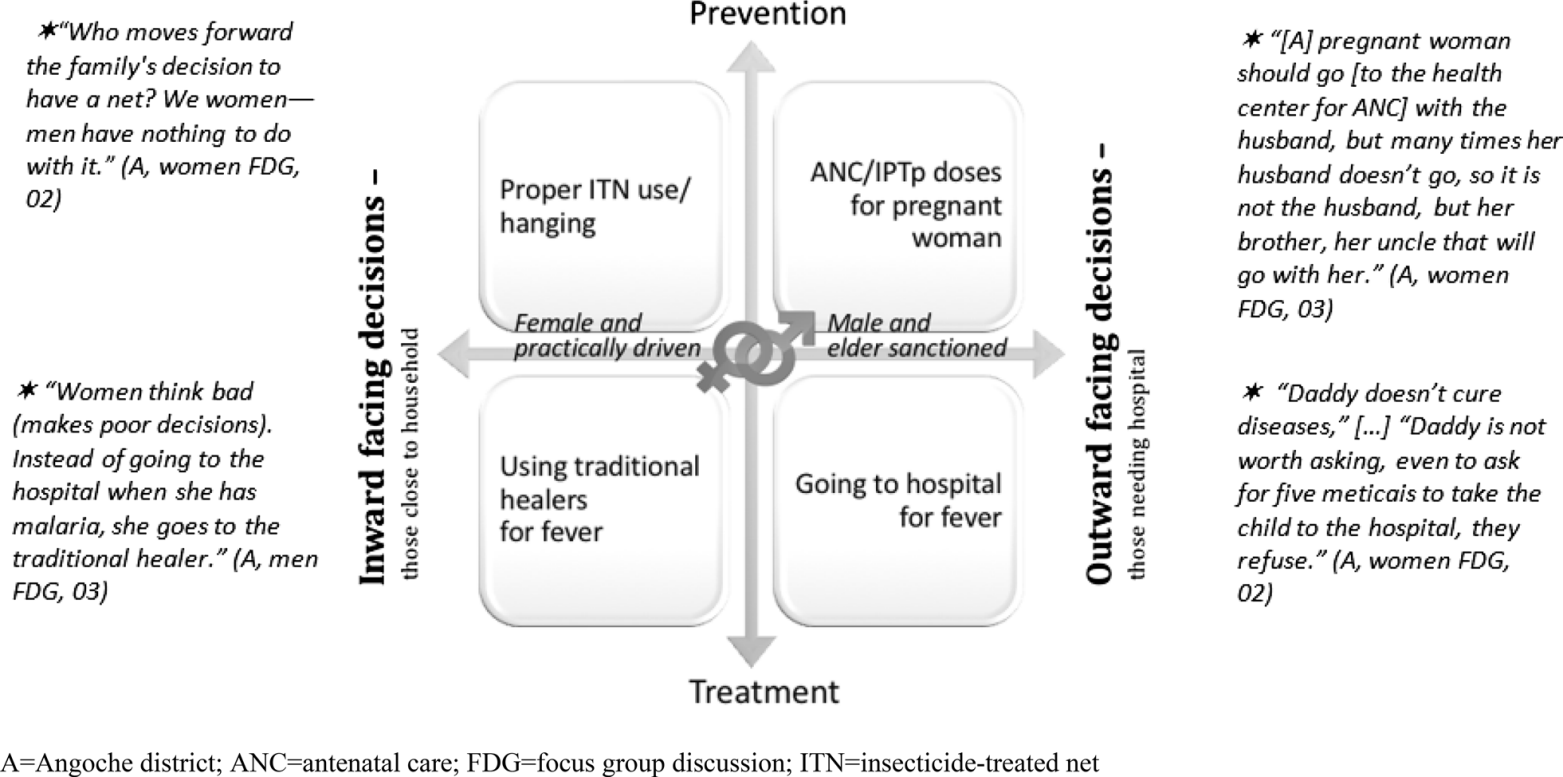
The gendered decision-making matrix for malaria prevention and treatment

Building on the above analysis, the study team extracted (5) a **gendered decision-making matrix** explaining emergent findings. This matrix was structured according to prevention or treatment behaviours and whether the prevention or treatment behaviours were inward or outward facing. Inward-facing behaviours relate to household-level factors under the influence of women, while outward-facing behaviours relate to activities away from the household that may require male and elder sanctioning. Outward-facing decision sanctioning was also sometimes under the purview of mothers-in-law or grandmothers, propagating hierarchical decision making within patriarchal systems, to which they themselves were also exposed during their own pregnancies. The gendered decision-making matrix is presented in Fig. 2. The illustrative quotes and sub-themes and related explanations anchoring them are described in turn.

#### Inward-facing prevention: proper use of bed nets

The proper use of nets was mostly seen as women’s business, although procurement of nets was not necessarily so. Proper use and care of nets was possible when women were given demonstrations regarding net importance and correct use as well as given access to nets, as evidenced by data from the TTSM intervention area. In both districts, registering the house for nets and collecting them during community distributions were reported to be the man’s task. In instances when there are not enough nets for all the household members and couples need to purchase additional nets, if they are available for sale, that purchase is the responsibility of the men:

Women *“don’t buy it, they don’t have money for that.”* (A, men FGD, 01).

Nevertheless, women repeatedly highlighted the notion that

“*Who moves forward the family’s decision to have a net? We women, men have nothing to do with it.”* (A, women FGD, 02).

Community actors reiterated this point:

*“In a couple, the element that demands the use of the mosquito net is the women.”* (A, IDI traditional healer).

This is partly maintained because the pregnant woman is the one who picks up the net from the health facility outside of the context of mass distribution campaigns. If the government distributes nets, women wait to get one. For instance, a woman from the comparison site noted that she is the one that must wait for the net because her husband

“doesn’t have the time.” (N, women FGD, 01).

Overall, since the women were mostly said to be the ones to collect the nets and men were mostly said to be working and outside the home anyway, the men rarely interfered with the decisions about net use. Concurrently, once the net was in the hands of women, unless men had been particularly sensitized to be supportive (e.g., by being tasked with repairs), they had little to do with how the nets were used. Although some shared decision making was needed at the point of entry into the house, in particular to convince men that gains could be had by proper use rather than reselling or using for agriculture, its subsequent daily use for malaria prevention was mainly an inward-facing prevention decision.

#### Outward-facing prevention: going to the facility for ANC/IPTp

Decision making for pregnant women going to the health facility for ANC/IPTp was consistently positioned as *an outward*-*facing prevention decision* that fell more under older and influential women to motivate but relied on men to fully sanction. Overall, many women claimed that men were difficult to persuade to endorse the decision, let alone accompany them:

*“Not all men go (with women to hospital for ANC checkups)* - *they are very rude men* - *[…] but when he is sick, I should go with him!”* (A, women FGD, 03).

Men often put men’s motivation to support these visits down to a desire for a check-up for other diseases, especially HIV. ANC was sometimes referred to as primarily for checking

*“the whole family […] if our bodies are okay.”* (N, men FGD, 01); and in particular for making sure the baby is doing well: *“because both men and women need to do tests and check if they have or do not have diseases to be able to have a healthy baby.” (*A, men FGD, 02).

There was an uncomfortable tension between health services providers’ recommendations to attend ANC with a partner and a few women in practice reporting feeling forced to do so to receive care, pushing some to “invent” replacement partners:

*“Because pregnant woman should go (to the health facility for ANC) with the husband, but many times her husband doesn’t go, so it is not the husband, but her brother, her uncle that will go with her.”* (A, men FGD, 03).

When asked, women mostly claimed they were accepting of IPTp, but men across the FGDs said women may fear going to the health facility for ANC, thinking they could be forced to take HIV medicine or cholera medicine. There was also a fear of delivery at the health facility due to rumours and consensus that some babies could have been mixed up at birth. These fears were said to at times push women to reject ANC and instead go to traditional healers. It was also consistently acknowledged that women felt most comfortable with the traditional healers because they knew them well, and felt they could trust them.

#### Outward-facing treatment: fast, effective care seeking at the health facility

Women throughout the FDGs explained that some fathers will accompany their children to the health facility, while others may just give money when a visit is necessary. Nevertheless, many women complained about their partners’ indifference and weak motivation to sanction health facility visits, as illustrated by one woman from the intervention site:

“*Daddy doesn’t cure diseases. […] Daddy is not worth asking, even to ask for five meticais* (Mozambique currency) *to take the child to the hospital, they refuse.”* (A, women FGD, 02).

As such, going to the health facility for malaria symptoms emerged as an outward-facing treatment decision prioritized by women, but under the decision-making authority of men and elders. As another woman put it:

“*Who initiates the conversations to take the child to the hospital? It is me […] the woman is the one that starts (the process of care seeking) because she has a mother’s feeling.”* (N, women FGD, 01).

#### Inward-facing treatment: care seeking from traditional healers

In contrast, women were often said to be the ones to make the decision to seek care from a traditional healer. One man asserted,

*“(A) woman thinks ‘badly’ (makes poor decisions), instead of going to the health facility when she has malaria; she goes to see a traditional healer.”* (A, men FGD, 03),

ignoring popular advice. Another explanation is that the option of a traditional healer is a more inward-facing treatment decision, allowing women to circumvent male sanctioning and seek the care options that are within their immediate control.

### Effects of the TTSM community dialogues

Major themes relating to the effects of TTSM community dialogues that emerged included the programme as a powerful (6) **source of change in relation to both perceptions of gender roles and how decisions are taken in the household**. These changes came from greater understanding and reported harmony within couples who agreed to share more of the domestic and household tasks. Changes were also put down to the sensitization of men, which aimed at normalizing their role as supporters of malaria prevention and treatment both inside and outside the home. In addition, there was (7) **strong community diffusion of TTSM messages**.

TTSM was viewed as a **source of change in relation both to perceptions of gender roles and how decisions are taken in the household**. The majority of participants in the programme activities identified TTSM with the changing roles and the promotion of a more egalitarian relationship between men and women. Significantly, the most widely retained TTSM message was that both men and women must participate in domestic chores:

“*The conversation of Tchova Tchova that I heard was that everybody must help at home*.” (A, women FGD, 01).

A consistent theme shared from a few of the men that participated in the programme was that these changes led to relationship “improvement”. These men talked about helping their wives with household chores and affirmed that it is not necessary to

*“wait for the woman to cook” because a man can “prepare a meal and save some for his wife to eat”* (A, men FGD, 01).

Some women that participated in the community dialogues emphasized equal rights between men and women:

*“Tchova Tchova says that women have rights, men have rights”.* (A, women FGD, 01).

In some cases, this message did lead husbands to start performing some of the household chores, not forcing their wives to do all these chores alone and expecting them to also go to the *machamba* (agriculture fields) to undertake heavy farming work on top of these existing tasks. For these women, this shift in expectations was experienced as a big and very welcome change:

*“I thank (the programme) because he does not leave all the housekeeping with me. He helps me.”* (A, women FGD, 01).

Nevertheless, changes that seek to modify cultural traditions are still sometimes difficult to assume in everyday life. As such, at times defence of the traditional gender roles was also stated by one man:

*“Here in Mozambique, men are at work and women at home.”* (A, men FGD, 02)

as he explained the gendered structure of activities. Another man claimed that rough treatment of women is for a good reason since it is up to men to assert dominance:

*“The pregnant woman cannot be offended (if treated roughly) even if she is your wife.”* (A, men FGD, 03).

This statement came from the intervention site, reflecting that not all men were convinced by TTSM messages, despite being sensitized to them. In the comparison non-intervention site, men’s and women’s traditional roles were also sometimes openly supported. For instance, when asked how to approach decision making to prevent malaria, men answered,

*“We remind our women to do the cleaning every day. To not leave pots with water for three, four days with dirty water.”* (N, men FGD, 02).

According to them, their women always follow these “commands”.

Correspondingly, women’s discomfort in addressing gender inequalities, and forcing discussions about health and care seeking in particular, was at times also notable, with some women themselves pointing to the discomfort of men who quit the programme:

*“They didn’t like this advice of helping women at home and they quit. It didn’t give them any advantage, that programme […] They say, ‘Help women? She is the one that married me!’.”* (A, women FGD, 02).

In contrast, there is no doubt that these participants felt that a harmonious relationship was fundamental to being able to share tasks, make joint decisions, and support each other’s health. Even in the nonintervention group, one woman responded proudly to the question

*“But what kind of husband goes with their wives to the hospital?” with “We have this, with my husband, we live in harmony.”* (N, women FGD, 01).

The concept of a harmonious household was a consistent thread throughout this study. Some men appeared to sincerely want to improve their relationships, help relieve their wives of household burdens, and make their homes peaceful and free of arguments. Accordingly, as participants shared their post-TTSM experiences, it also emerged that the intervention had positive effects beyond changing attitudes and sparking more discussions. From men’s narratives, it emerged that they not only wanted to be more supportive of prevention and treatment seeking for family members because of the sessions but had also been prompted to abandon the idea that men are always strong and never must look for help themselves:

*“A long time ago, before participating in the (community dialogue) sessions with the facilitators, when I felt malaria symptoms, I preferred to go for a run to get rid of the pains, but I didn’t know what I was doing […] I shouldn’t be doing it, I should go to the hospital.”* (A, men FGD, 03).

The acceptance of men bringing their sick children to the health facility or accompanying their pregnant wife to her first appointment was presented as a positive change toward a more harmonious relationship:

*“Men did not like to take the children to the hospital; they had the idea that only women should do that. But now with this ‘Stop Malaria’ (TTSM), men change their minds about this.”* (A, men FGD, 02).

Overall, doing things together as a couple had a unifying effect, such as sharing a bed net:

*“In my case, we slept separated, and when we got the net, we sleep together; it made us closer.”* (A, women FGD, 03). And among women:

*“We all use the nets and share our secrets on this.”* (A, women FGD, 01).

In cases in which men were said to be unsupportive, women talked about living in a fractious home environment characterized by abuse, jealousy and husbands denying paternity.

A notable sub-theme that resulted from **community diffusion of TTSM messages** was the community “taking action” to help neighbours make the “right” decisions. A few participants expressed the following sentiment:

*“If we see that our neighbour’s patio is cluttered, we advise them to tidy it to prevent malaria, and then we inform them that clutter creates (potential for standing water and can lead to) breeding sites for malaria.”* (A, women FGD, 01).

These community actions also explained how some messages had become so diffused:

“*We get out and go to the neighbour’s house. When we get there, and we find someone with malaria, we advise them to take the child to the hospital. We inform them that malaria is not cured at home, only at the hospital.”* (A, women FGD, 01).

Sleeping under the net became part of the *shared discourse of the TTSM participants* because not only were they told of its benefits but also because both women and men said that, thanks to the programme, they had learned how to use their nets properly (hanging, washing, repairing, etc.). The demystification of net use thanks to TTSM was discussed across FDGs, for instance:

*“To me it helped because before I didn’t use (the net) because of fear, but now I sleep under the net with my kids, and I like it.”* (A, women FGD, 03).

Another important sub-theme about diffusion was that it consistently emerged that intervention participants needed reassuring that it is acceptable to share knowledge about something you have not experienced, and despite not having experienced it you can still learn about it and can be confident to share that knowledge. It appeared as a cultural barrier that knowledge without experience is considered *fofoca* (irrelevant gossip).

Overall, TTSM encouraged participants to override the rules about who can say what, making programme messages the topic, rather than focusing on personal experiences, and enabling better communication between neighbours. This collective shift in ways of communicating was shown to be a powerful tool for change.

## Discussion

The main findings from this study on malaria-related decision making in rural Nampula Province of Mozambique underscore the role of social norms and community, family hierarchy and gendered power dynamics in influencing behaviour. The introduction of the gendered decision-making matrix, organized by inward- and outward-facing behaviours, contributed to a clearer understanding of gendered decision-making pathways. In other African contexts, care-seeking decisions are also likely to be determined by a sequence or chain of decisions in which women often have little or no control, with men typically weighing in on the final decision, particularly if services require a monetary payment [34, 35].

This study found that a majority of intervention participants considered TTSM to be a helpful because it promoted relationship shifts toward a more egalitarian, harmonious household. The programme was also said to contribute to changing malaria prevention and treatment behaviours and attitudes that support them. In particular, this study found that TTSM affected how decisions are made in the household, encouraging more partner collaboration in many areas of life. While making decisions about malaria-related practices remained a complex process, both men and women who participated in TTSM referred more often, relative to the comparison group, to sharing of household chores, sharing nets, valuing shared decision making, and having male involvement in care seeking for malaria.

Nevertheless, the positive context of a strong, balanced and harmonious partnership between men and women was recognized and celebrated in both study settings. The concept of the harmonious household was notable in Mozambique in early gender-related studies by the Center for Communication Programs [36]. It was also identified in other African settings, such as Kenya [37], which similarly coined the term and identified its importance in daily life and couple communication relating to family planning. In Senegal, this concept was named as defining the opposite end of the spectrum to gender-based violence [38]. Leveraging the harmonious household concept appeals to both men’s and women’s aspirations and desires. Still, men themselves experience particular dilemmas in taking responsibility not just for their families, but also for their own health.

It transpired in this study, and is also well known, that structural gender norms have long upheld the notion that men must be “strong” and are less vulnerable to diseases than women and children. Moreover, frequent accessing of health facilities is regarded as typical for women, but not for men [39]. TTSM’s addressing the need for men to also seek care for malaria led to men discussing positive changes, such as no longer trying to simply ‘sweat out’ the disease through vigorous exercise, but getting proper treatment instead. Therefore, men need to be encouraged in health care seeking for themselves, as well as in supporting their families in this area. Using respected male role models in an effort to reach men more meaningfully will serve future gender-sensitive programmes well [40].

Nevertheless, including men should not happen in the absence of gender synchronization. That is, working with men and women should happen in tandem and not in a silo-ed manner [41] because the latter approach has had mixed success. As such before making programmatic recommendations, future programme managers should explore when and under what circumstances it is useful to encourage men to accompany their partners or wives to the clinic, taking into consideration their life circumstances, resources needed and evidence for the benefits of partner presence during such visits. Evidence that women feel that attending ANC with a partner is a “requirement” to receive care shows how policies intended to support one goal (i.e., HIV and STI testing) can have unintended and detrimental effects for another.

The current study shows that it is vital to continue to support and encourage care seeking by single mothers, who are the most vulnerable to neonatal fatalities [42]. Messages emphasizing prompt care seeking at the facility for fever also need to emphasize *supporting and enabling women to get there in the first place*, circumventing time-consuming, outward-facing negotiations that prevent women from accessing timely care. Women may attempt to offset these vulnerabilities by reaching out to their social networks. Traditional birth attendants and healers play important roles in women’s inward lives. In Zambia, for instance, these trusting relationships have been shown to often be preferred by women in relation to their reproductive health [43]. In Kenya, such traditional services have been found to be more accessible because deferred payments or payments in kind may be possible [44], perhaps with produce or gifts that women make themselves.

For the most vulnerable and oppressed women, building capacity for traditional birth attendants and healers, as well as ambulant services, to bring information, diagnostics and treatment into the home is essential. For example, a study conducted in Bogoma, Kenya, concluded that it is women, not men, who have the most difficulty in recognizing malaria symptoms. The study also found that symptoms such as seizures and high fever may be attributed to supernatural causes by the community, rather than malaria. Such beliefs may further encourage some women to look for a traditional healer before seeking health care services [45, 46].

A well-rounded communication campaign should include information for traditional healers and community health workers alike. Additionally, it would depict gains that can be attained by changing behaviour, yielding rewards for both men and women, and depicting explicitly what these simple changes can bring. In particular, the notion of the harmonious household should be harnessed to enhance and encourage better decision making, especially in already stable relationships. Other key messages that should be incorporated into future messaging include the time- and money-saving advantages of malaria prevention, including avoiding illness and time out of work and promoting wellness, among others.

## Strengths and limitations of the study

This study considers not only the malaria-endemic context, for which much is descriptively known, but also the cultural and psycho-social influences that are crucial to behaviour change yet have to date been under-researched in relation to gender and social norms. This study utilized a comparative approach, illustrating consistent themes across intervention and non-intervention sites in relation to gender norms and aspirations, and provided evidence on TTSM effects by exploring perspectives particular to the intervention setting, using a gender-sensitive lens. This study does not however examine significant difference in exposed or unexposed groups, this would require a quantitative study design, ideally with a longer time lapse after exposure, which is recommended for future directions of TTSM-related research.

Analysis is also limited to the defined study populations, and by translated data in ‘expanded note’ form, that may have masked nuances in meaning and hindered more in-depth analysis. While the study achieved saturation on major themes, it is recommended that future studies explore in greater detail supporting sub-themes related to the gendered decision-making matrix, such as the dynamics that underpin it, and contexts in which it is applicable, or in which it can be differently configured.

## Conclusions

This study, guided by the proposed gendered decision-making matrix, contributes to a better understanding of men’s and women’s decision making regarding the prevention and treatment of malaria, in part by highlighting inward- and outward-facing behaviours. This helps identify where gendered pathways could be interrupted, redirected or, if health promoting, supported. Overall, these data suggest that the matrix can help programme designers and implementers as well as participants recognize and support positive factors and challenge negative ones.

The focus on the inward/outward gendered structuralizing of decision making highlighted the central need to counteract men’s underexposure and traditional lack of involvement in public health programmes in the Mozambique context. Given that similar situations are likely to be present in many other African settings [47], field testing this matrix in other countries and adapting it as needed would be useful.

This study also provides evidence regarding the ameliorating effects of participation in TTSM dialogues on malaria-related household decision making. The results should be used to inform both community-based strategies and mass media programming to improve malaria prevention and treatment. These insights suggest that media campaigns could promote harmonious gender-equal households by modelling couple communication and shared decision-making, which would contribute to improved malaria-related and other health-related outcomes in Mozambique and beyond.

## Data Availability

Data and materials are not available for public access. The data repository is held at Johns Hopkins Center for Communication Programs.

## Abbreviations

A: Angoche
ANC: Antenatal care
CCP: Center for Communication Programs
FGD: Focus group discussion
HC3: Health Communication Capacity Collaborative
IDI: In-depth interview
IPTp: Intermittent preventive treatment in pregnancy
ITN: Insecticide-treated net
N: Nacaroa
TTSM: *Tchova Tchova* Stop Malaria
